# Videoconferencing Delivery of the Seoul Premarital Education Program During COVID-19: A Quasi-experimental Study Using Inverse Probability of Treatment Weighting

**DOI:** 10.1007/s11121-024-01761-z

**Published:** 2025-01-06

**Authors:** Jisu Park, Jaerim Lee

**Affiliations:** 1 Department of Child Development and Family Studies, College of Human Ecology, Seoul National University, 1 Gwanak-Ro Gwanak-Gu, Seoul, Republic of Korea; 2 Research Institute of Human Ecology, College of Human Ecology, Seoul National University, Seoul, Republic of Korea

**Keywords:** Couple relationship education, Family life education, Interactive videoconferencing, Internet-based prevention, Online implementation, Web-based intervention

## Abstract

This study examined whether the Seoul Premarital Education Program (S-PEP) was effective when it was delivered via videoconferencing (VC) during the early COVID-19 period. S-PEP is a city-wide educational program designed to prevent marital distress by enhancing premarital readiness and relationship quality for couples. Because a randomized controlled trial was not realistic due to the pandemic, we recruited an intervention group and a no-intervention comparison group independently, who completed both the pre- and posttests. We then employed inverse probability of treatment weighting to derive two comparable groups. The weighted samples of 291 women and 228 men were analyzed separately using linear mixed models. The results showed that S-PEP via VC was effective in increasing levels of marital readiness for both women and men and in enhancing relationship confidence and satisfaction for women. Our findings suggest that VC can be a promising delivery strategy for curriculum-based group interventions for couples.

**Supplementary Information:**

The online version contains supplementary material available at 10.1007/s11121-024-01761-z.

Countries like South Korea (Korea hereafter) have taken preventative approaches to respond to marital distress by offering educational programs for premarital couples at community-based family service agencies (Lee & Son, 2018). Although the literature has documented that premarital education can prevent relationship distress by improving relationship skills and quality (Carroll & Doherty, 2003; Hawkins et al., 2017; Rhoades, 2015), the population-level impact of these programs has been limited because a relatively small number of couples have participated (Clyde et al., 2020). It is crucial to increase participation by expanding delivery modes beyond traditional in-person methods (Doss et al., 2016).

Online preventive interventions have received considerable attention over the past decades thanks to the accessibility of asynchronous, self-directed online courses. The literature on couple relationship education (CRE) has shown that self-directed online CRE programs such as ePREP, an online format of the Prevention and Relationship Education Program (PREP) (Braithwaite & Fincham, 2007), and OurRelationship, a web-based CRE curriculum (Doss et al., 2013), are successful in reaching diverse couples (Georgia Salivar et al., 2018). They have also been found to be effective in enhancing the quality of couple relationships and their physical and mental health (Doss et al., 2020; Roddy et al., 2020). However, self-directed online interventions are restricted because they are mostly based on informative web pages and apps without synchronous interactions between facilitators and participants.

Videoconferencing (VC) is a promising alternative to asynchronous, self-directed online interventions. VC enables practitioners to interact with multiple participants and to monitor their reactions synchronously (Correia et al., 2020; Darling et al., 2022). VC existed before COVID-19, but VC-delivered CRE became widespread during the pandemic when VC dramatically expanded as a major medium for group-based interventions under social distancing restrictions. It is very likely that preventive interventions via VC will remain a prominent method of delivery for CRE and other prevention programs after the pandemic (Markman et al., 2022). However, little is known about whether CRE via VC can lead to the intended outcomes, especially in non-Western contexts. The purpose of this study is to examine whether the Seoul Premarital Education Program (S-PEP) was effective in increasing marital readiness, relationship confidence, and relationship satisfaction among Korean premarital women and men when the program was delivered via VC during the early COVID-19 period.

S-PEP is a preventive curriculum that has been implemented throughout Seoul since 2015. It is funded by the Seoul Metropolitan Government. Because it was not plausible to employ a randomized controlled trial (RCT) during the early pandemic, we recruited an intervention group and comparison group separately and employed inverse probability of treatment weighting to obtain two comparable groups of premarital individuals. This study is expected to contribute to the literature on online CRE and other online preventive interventions.

## Background

### Premarital Education as a Meaningful Preventive Intervention in Korea

Premarital education is a type of CRE that provides “structural learning experiences to help couples develop their relationship knowledge, attitudes, and skills” (Halford, 2011, p. 2). Premarital education, also known as marriage preparation interventions or premarital prevention, refers to a professional practice designed to help individuals and couples prepare for a healthy marriage using an educational, preventive, and strengths-based approach (Clyde et al., 2020; Duncan et al., 2010). Premarital education aims to prevent marital distress by equipping individuals and couples with the knowledge and skills needed for a healthy marital relationship and effective functioning (Clyde et al., 2020). When widely targeted, premarital education is offered to unmarried individuals and couples in a wide range of developmental stages starting in adolescence. When narrowly targeted, premarital education focuses on couples who are engaged or are considering marrying the current partner, which is the case for S-PEP in this study. The target audience of S-PEP was selected because a prevention program is more likely to be effective when it addresses highly relevant topics for the intended audience (Small et al., 2009).

Premarital education has unique preventive significance (Clyde et al., 2020). According to family development theory (Rodgers & White, 2009), the transition to marriage involves new developmental tasks as a couple and requires dyadic adjustment to the new family life stage. Couples tend to experience substantial changes during this transition including establishing new roles, rules, and routines as a married couple (Hall & Adams, 2020). Because of these changes, getting married is a major stressful life event (Holmes & Rahe, 1967) from a family stress perspective. In countries like Korea where non-marital cohabitation is uncommon and domestic partnerships are not legally acknowledged, couples typically move in and begin to share resources and experience daily adaptations during the transition to marriage.

The transition to marriage is often a gendered experience for heterosexual couples. Research has shown that women and men tend to have different attitudes toward and expectations about marital life, particularly in countries with traditional gender roles such as Korea (Yoo, 2020). Dissimilar attitudes may lead to difficulty with dyadic adjustment and lower relationship quality (Arránz Becker, 2013; Gaunt, 2006; Keizer & Komter, 2015). The gendered nature of marriage (Bernard, 1982) suggests that gender should be considered in premarital education and examined in related research.

Korea is unique in its approach to preventive interventions for couples and families. Based on the Framework Act on Healthy Families of 2004, central and municipal governments fund and oversee the community-based Family Centers that provide family services such as free premarital education (Lee & Son, 2018). The Framework Act signified a paradigm shift of Korean family policy from a selective, problem-solving approach to a comprehensive, preventive approach (Chin et al., 2012). As of 2023, 211 local Family Centers serve individuals and families throughout the nation (Ministry of Gender Equality & Family, 2023). In Seoul, all 25 districts have their own Family Centers, and one city-level headquarters oversees the 25 Centers.

### Seoul Premarital Education Program (S-PEP): A City-Level Preventive Curriculum

S-PEP is one of five Seoul Family School programs that have been implemented at local Family Centers since 2015. With the funding and oversight of the Seoul Metropolitan Government, the Headquarters of Seoul Family Centers designed and disseminated the S-PEP curriculum in collaboration with family scholars and practitioners. The Headquarters of Seoul Family Centers selects and trains qualified S-PEP instructors who deliver lectures and facilitate activities. The instructors are required to adhere to the instructor’s manual and to utilize the prescribed teaching materials.

The objective of S-PEP is to enhance marital readiness and relationship quality, and it is expected to prevent marital distress and maladjustment once a couple is married. S-PEP helps premarital couples build knowledge and skills for a healthy marriage by providing opportunities to discuss issues related to marital life. The target audience is couples who are engaged or are considering marrying the current partner. This audience was selected instead of all premarital individuals regardless of partnership status because couples who are close to getting married may find S-PEP much more relevant to their real life.

S-PEP has evolved since it was first implemented in 2015. In 2020, the S-PEP curriculum included six modules on six major domains of marital life: (a) understanding each other’s differences, (b) couple communication, (c) the meaning of marriage, (d) financial management, (e) gender equality and division of housework, and (f) life histories and families-of-origin. The Centers typically selected four of the six modules with one session for each module. The sessions were offered on two consecutive Saturdays with two sessions (modules) per day. Each module was a 2-h group session with lectures, small group discussions, skills training, and checklists. Registration is free of charge, and couples are instructed to attend all sessions together.

S-PEP was developed as an in-person intervention but had to be delivered via VC during COVID-19. This VC-delivered S-PEP adhered to the program manual including the contents and length of instruction and couple activities. In most cases, each couple was in the same location using one smart device or computer when they attended S-PEP via VC, which enabled them to participate in the couple activities face-to-face while also being virtually connected to other couples and facilitators. The Headquarters of Seoul Family Centers tallied the total number of attendants for each session of the S-PEP and counted the same individuals multiple times. This resulted in 6407 counts in 2018, 6456 in 2019, and 4263 in 2020 (Lee et al., 2020; Seoul Healthy Family Support Center, 2020). Considering that S-PEP consists of four sessions on average, we can interpret that approximately 1600 individuals attended S-PEP each year in 2018 and 2019. In 2020, roughly 1000 individuals participated in S-PEP via VC offered by 25 Family Centers.

Surprisingly, no evaluation of the efficacy and effectiveness of S-PEP (in-person or VC) has been published in scholarly journals (Lee & Son, 2018; Lee et al., 2020). Only satisfaction surveys and anecdotal testimonies have been common practices. Given the several years of implementation and the substantial number of participants, it is imperative and urgent to examine the effectiveness of S-PEP to move toward an evidence-based program.

### Couple Relationship Education via Videoconferencing

Internet-based CRE has increased given that it can overcome geographic barriers and reach more couples living in an overscheduled, fast-paced world (Barton et al., 2020; Hughes et al., 2012). The efficacy and effectiveness of online CRE programs such as ePREP (Braithwaite & Fincham, 2007) and OurRelationship (Doss et al., 2013) have been documented (Roddy et al., 2020). For instance, an early study reported that the effects of online CRE were equivalent to in-person CRE (Duncan et al., 2009). A recent meta-analysis also showed significant effects of online CRE on couple relationships and individual well-being (Megale et al., 2022).

Previous studies on online CRE have focused heavily on asynchronous, self-directed courses with activities such as reading articles, watching videos, answering quizzes, and responding in writing to discussion questions. However, self-directed online CRE is restricted because it lacks real-time interaction, which makes it difficult for participants to stay motivated and engaged (Zemp et al., 2017). This limitation is a threat to retention over time. As an alternative, recent studies have introduced a blended approach that adds synchronous components to self-directed online CRE, such as phone or video sessions with practitioners (McAllister et al., 2012; Roddy et al., 2018). These studies have shown that blended programs are more effective in enhancing couple relationships than self-directed CRE alone (Halford et al., 2010; McAllister et al., 2012; Roddy et al., 2018; Zemp et al., 2017). For example, OurRelationship resulted in greater reduction in anxiety when it was combined with a higher level of coaching compared with a lower level of coaching (Roddy et al., 2018).

VC has emerged as an attractive alternative to in-person interventions, particularly during COVID-19. VC enables real-time virtual interaction by sharing video, audio, and screens and utilizing interactive tools such as chats, polls, and breakout rooms. VC has dramatically changed how online interventions are delivered (Dadds et al., 2019; Estrada et al., 2019; Gentry et al., 2018) and has been found to be an effective platform (Gentry et al., 2018). The use of VC in CRE is relatively recent (Tsai et al., 2020; Turner et al., 2022), but it is expected to remain prevalent (Markman et al., 2022). However, little empirical research has examined whether preventive group interventions via VC are effective, and few studies have examined relationship education programs via VC (Turner et al., 2022).

### The Current Study

The purpose of this study is to test whether participation in S-PEP increased marital readiness, relationship confidence, and relationship satisfaction for premarital women and men when the program was delivered via VC during the early COVD-19 period. The data for the present study came from a research project that aimed to evaluate the effectiveness of S-PEP for the first time. The research project was set to launch in March 2020, which, unexpectedly, was the early COVID-19 period. This unprecedented timing offered us an opportunity to innovatively test VC-delivered S-PEP, but a RCT was unrealistic during the early pandemic. It was not feasible to randomly assign enrolled participants to a comparison group because local Family Centers needed to maintain enough participants for funding purposes, even though recruitment was challenging. Thus, we employed a quasi-experimental design with a separately recruited comparison group. Specifically, we used a pre-post comparison design with a nonequivalent comparison group (Handley et al., 2018) along with inverse probability of treatment weighting to analyze equivalent intervention and comparison groups. Our overarching research question was whether pre-to-post changes in marital readiness, relationship confidence, and relationship satisfaction significantly differed between the intervention group and the no-intervention comparison group of premarital women and men.

We examined the pre-to-post changes in marital readiness, relationship confidence, and relationship satisfaction. These dependent variables were selected because S-PEP aims to prevent marital distress (a long-term outcome) by enhancing marital readiness and relationship quality for premarital couples (short-term outcomes). Given our pre-post comparison design, we focused on the intended short-term outcomes of S-PEP. Marital readiness, our primary outcome measure, refers to an individual’s level of preparedness to maintain a healthy marriage, which includes knowledge, skills, and behaviors across major domains of marital life (Lee & Park, 2021). In this study, these domains encompassed the following: marriage-related values, goals, and expectations; couple communication; sexuality; family planning and parenthood; relationships with each partner’s families-of-origin; financial management; work-family issues and division of housework. Prior research that has conceptualized and operationalized marital readiness has shown that these domains are culturally relevant for Korean premarital couples (Kim & Shin, 2002; Lee, 2003; Lee & Park, 2021).

Relationship confidence and relationship satisfaction served as additional outcome measures to assess the short-term effects of S-PEP. Relationship confidence reflects an individual’s belief in the couple’s ability to maintain a healthy, stable, romantic relationship by effectively managing future conflicts (Whitton et al., 2007). Studies have shown that higher relationship confidence correlates with greater marital satisfaction (Johnson & Anderson, 2013) while lower relationship confidence is associated with increased marital risk factors (Allen et al., 2010; Kline et al., 2004). Finally, relationship satisfaction, a key aspect of relationship quality, has been widely documented in previous research (Hendrick et al., 1998).

## Method

### Procedures and Participants

**Fig. 1:**
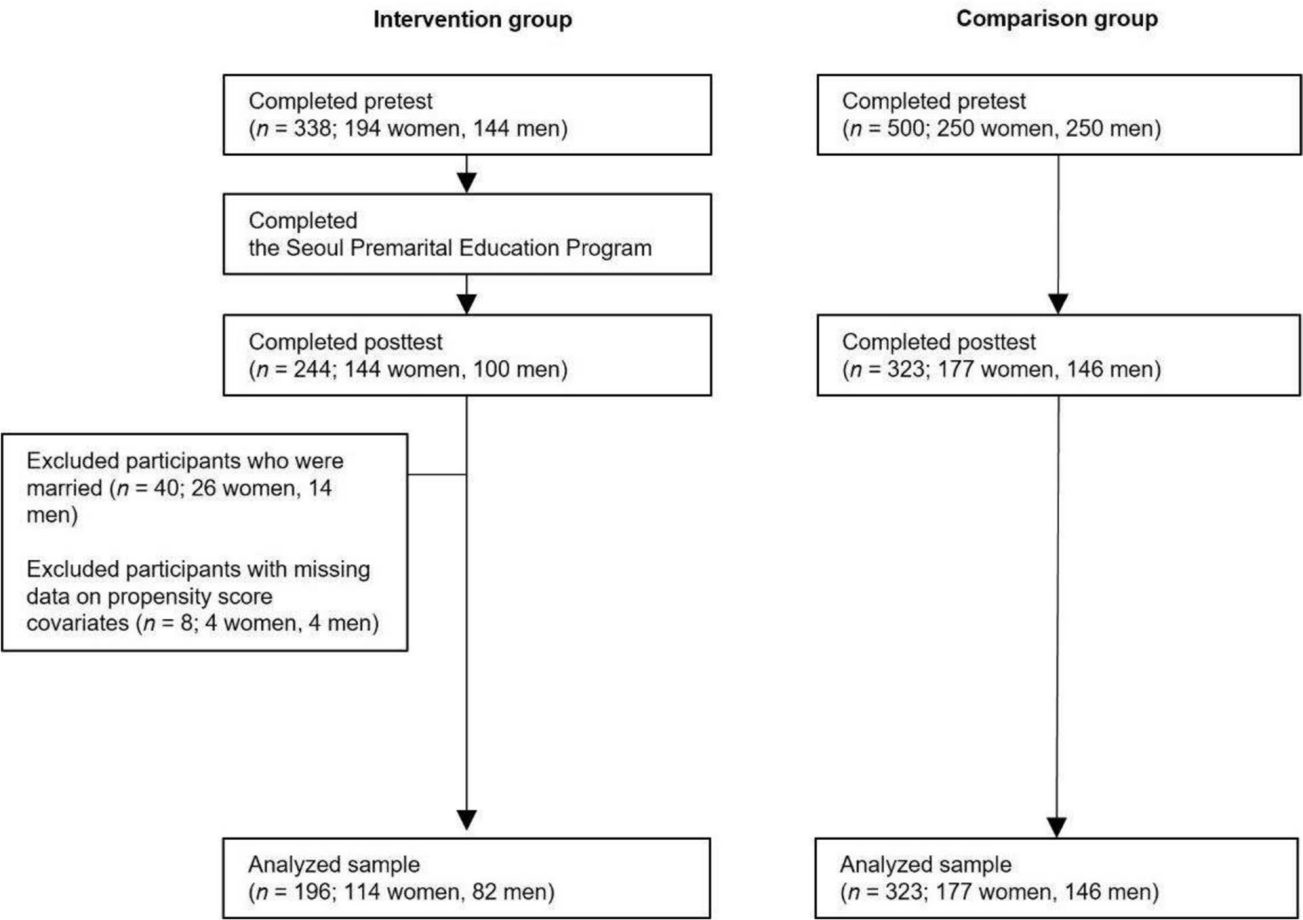
CONSORT flowchart of participants

Figure 1 displays the CONSORT flowchart of participants. The research project was approved by the university IRB of the authors’ institution. The authors did not have a conflict of interest with S-PEP.

#### Intervention Group

The intervention group was a sample of individuals who participated in one of the 21 S-PEP groups between June and October 2020 when Family Centers reopened and started to offer all group sessions virtually due to social distancing restrictions. Given the sudden change in the delivery mode and lack of experience with online group interventions, local Centers were allowed to select a VC platform that adequately served their communities. Zoom was the most commonly selected platform followed by Gooroomee, a Korea-based VC platform. We excluded groups that did not use VC such as prerecorded or live streaming video services like YouTube.

Our recruitment flyer was sent to all individuals who enrolled in one of the 21 S-PEP groups offered by the 17 Family Centers that agreed to participate in our research. Individuals who voluntarily provided online consent were directed to the pretest Qualtrics page, to which they responded using their smart devices or computers. A total of 338 individuals completed the pretest and then attended the first session of their group. Of the 338 individuals, those who attended the final session of their group were invited to complete the online posttest. Of the 338 pretest respondents, 244 completed the final session of the intervention and responded to the posttest. Both the pretest and posttest were conducted in Korean. Those who completed both tests received a coffee gift card.

For the following analysis, we selected participants who completed both the pre- and posttests and were not married at pretest, which resulted in a final sample of 196 individuals (114 women, 82 men). Participants who dropped out after the pretest did not differ statistically from those who completed both tests regarding our covariates and dependent variables except for having a shorter relationship (*t* = 5.45, *p* < 0.001). Married participants at pretest were excluded given that the target audience of S-PEP was premarital couples. We also excluded participants with missing information on the covariates used to calculate propensity scores. The unit of participation in S-PEP was a couple, but the unit of our sampling was an individual for both the intervention group and the comparison group. The intervention group sample comprised 56 couples (112 partners) and 84 individuals who completed S-PEP as a couple but whose partners were not included in the data due to non-response to either the pretest or posttest. We found no statistical differences between the 112 partners and the 84 individuals. No same-sex couples participated.

We checked adherence fidelity of implementation to ensure that the 21 S-PEP groups were exposed to the same curriculum contents and coverage despite the sudden change of delivery mode to VC. Using an online fidelity checklist, staff monitoring the 21 groups responded to questions about whether each session included the prescribed lectures and activities in the S-PEP manual. The fidelity score of each session was 86.94 out of 100, on average (range 72–95).

#### Comparison Group

The comparison group was independently recruited in December 2020 from a massive panel of Macromille Embrain, a leading online survey company in Korea. To construct a comparison group with demographic characteristics equivalent to the intervention group, we set the following selection criteria: individuals who (a) lived in Seoul, (b) were 25–44 years old, (c) were never married, (d) were in a committed relationship, and (e) intended to marry the current partner (4–5 out of 5). These criteria were chosen based on the descriptive characteristics of the intervention group. We used quota sampling that required equal distribution of women and men, but they were not matched couples.

From the massive online panel, randomly selected individuals who met our selection criteria received an invitation to participate in our pretest via a text message and an email. Those who voluntarily provided online consent were allowed to complete the pretest via their smart devices or computers. We stopped data collection when we reached a sample of 250 women and 250 men given our limited budget. After two weeks, the same interval between the pretest and posttest for the intervention group, all 500 individuals received an invitation to complete the posttest. A total of 323 individuals (177 women, 146 men) completed the posttest, which became our comparison group.

### Measures

#### Dependent Variables

We used the 28-item Korean Marital Readiness Questionnaire (Lee & Park, 2021) to assess marital readiness. This measure was developed by modifying earlier measures of marital readiness in Korean (Kim & Shin, 2002; Lee, 2003) and covers major domains of marital life that are culturally relevant. Sample items are “I know what kind of marriage my partner desires,” “We discussed how to manage our finances when we are married,” “I can easily talk about the issues on which my partner and I have different opinions,” and “We discussed how to divide housework” (1 = *strongly disagree*, 5 = *strongly agree*). A higher score indicated a higher level of marital readiness. Cronbach’s alphas for the 28 items were 0.91 for the intervention group and 0.92 for the comparison group at pretest.

The 4-item Relationship Confidence Scale (Stanley et al., 1994) was used to measure relationship confidence. Sample items include “I believe we can handle whatever conflicts will arise in the future” and “I am very confident when I think of our future together” (1 = *strongly disagree*, 5 = *strongly agree*). A higher score indicated a higher level of relationship confidence. Cronbach’s alphas were 0.90 for both the intervention and comparison groups at pretest.

We used the Relationship Assessment Scale (RAS; Hendrick et al., 1998) to assess relationship satisfaction with the current partner. This 7-item scale measures overall relationship satisfaction using 5-point Likert scales (vary by question). Sample items are “In general, how satisfied are you with your relationship?” and “How well does your partner meet your needs?” A higher mean score indicated a higher level of relationship satisfaction. Cronbach’s alphas for the seven items were 0.88 for the intervention group and 0.85 for the comparison group at pretest. We used arithmetic means for marital readiness, relationship confidence, and relationship satisfaction for the analysis.

### Analysis Strategy

We first used inverse probability of treatment weighting (IPTW) to adjust for dissimilarities in the background characteristics between the S-PEP intervention group and the no-intervention comparison group. IPTW, an extension of the propensity score methods, statistically adjusts differences between two groups by weighting each unit using the inverse of the probability of receiving the treatment (Austin, 2011; Rosenbaum & Rubin, 1983). IPTW is preferred over propensity score matching that loses unmatched cases. We then employed linear mixed models to estimate the treatment effect using a balanced sample obtained through IPTW. In this study, we analyzed women and men separately so as not to violate the independence assumption given that our intervention group data included couples. A dyadic analysis was not adequate because our no-intervention comparison group did not include matched couples.

The first step of IPTW involves computing propensity scores, which represents the probability of being assigned to the treatment group based on covariates measured at the pretest. These covariates should be chosen based on the literature and practical knowledge (Guo & Fraser, 2014). We carefully reviewed previous studies that examined the characteristics of individuals who seek help with their romantic relationships through educational interventions compared to those who do not. The individual characteristics that these studies have reported include age (McAllister et al., 2013), education (Schofield et al., 2015; Stanley et al., 2006), and socioeconomic status (Adler-Baeder et al., 2010; Schofield et al., 2015; Stanley et al., 2006), along with religious and attitudinal perspectives on marriage (Blair & Cordova, 2009; Duncan et al., 2007). Couple characteristics such as the duration of the relationship (Park et al., 2022) and the level of negative couple interaction (Duncan et al., 2007; Morris et al., 2011) have also been identified as predictors of participation in CRE programs. As a result of the thorough literature review, we calculated the propensity scores using age, education, income, subjective socioeconomic status, religion, length of the current relationship, attitude toward getting married, and negative couple interaction as covariates. Supplementary Table 1 online shows how these covariates were measured.

The next step of IPTW was to assign a weight of “1” to the intervention group and a weight of “1 minus the inverse of propensity score” to the comparison group. To ascertain whether this adjustment using IPTW successfully resulted in group equivalence on the modeled covariates, we evaluated the balance using standardized mean differences (SMDs) between the covariates of the two groups. Supplementary Table 4 online presents the descriptive statistics of the covariates and the SMDs before and after IPTW. The absolute values of all SMDs after IPTW were less than 0.25, and the variance ratios were below 2.0, which indicates that our IPTW models were adequately specified. The statistical analyses involving IPTW were conducted using R software, version 4.4.0 and followed Baek and Park’s (2021) guidelines.

We employed linear mixed models using the weighted samples because our data were nested within individuals due to repeated measures. We estimated 2-level models using the R lme4 package to account for the nesting of individual observations (level 1) within individuals (level 2). We included intervention-by-time interaction terms to examine whether pre-to-post changes in marital readiness, relationship confidence, and relationship satisfaction differed by intervention participation. We controlled for the eight covariates used in IPTW to consider group differences that may remain after IPTW. Supplementary Table 2 and Table 3 online present the correlation matrix for all variables included in our linear mixed models.

## Results

**Fig. 2:**
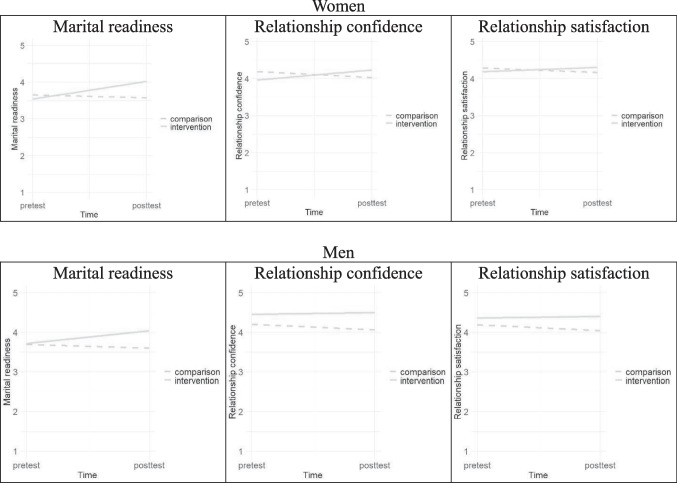
Intervention-by-time interaction for the outcome variables by gender

Table 1 and Fig. 2 show the results of our linear mixed models. In our models, estimates for the intervention variable represented differences between the intervention group and the comparison group at pretest. Estimates for the time variable represented pre-to-post changes in the outcome variables, and estimates for the intervention-by-time interaction term represented differences in the slopes between the two groups. Significant interaction indicates that the effect of S-PEP on pre-to-post changes was significant in the outcome variables.

**Table 1:** Fixed effects results from the linear mixed models

			Women (*N* = 291)			
	Marital readiness		Relationship confidence		Relationship satisfaction	
Variable	*B*	*SE*	*B*	*SE*	*B*	*SE*
Fixed effects						
Intercept	3.98^***^	0.77	4.71^***^	0.92	4.66^***^	0.80
Intervention ^a^	−0.07	0.06	−0.14	0.08	−0.01	0.07
Time ^b^	−0.08^**^	0.03	−0.18^***^	0.04	−0.13^***^	0.03
Intervention by time	0.56^***^	0.05	0.49^***^	0.07	0.25^***^	0.06
Random effects						
Intercept variance (*SD*)	0.18 (0.43)		0.24 (0.49)		0.18 (.43)	
Residual variance (*SD*)	0.10 (0.32)		0.21 (0.45)		0.15 (.38)	
−2log likelihood	707.5		1020.0		839.1	
			Men (*N* = 228)			
	Marital readiness		Relationship confidence		Relationship satisfaction	
Variable	*B*	*SE*	*B*	*SE*	*B*	*SE*
Fixed effects						
Intercept	3.37^***^	0.79	3.83^***^	0.87	4.69^***^	0.79
Intervention ^a^	0.04	0.07	0.26^**^	0.09	0.21^**^	0.08
Time ^b^	−0.09^**^	0.03	−0.14^**^	0.05	−0.15^***^	0.04
Intervention by time	0.42^***^	0.06	0.19	0.10	0.19^*^	0.08
Random effects						
Intercept variance (*SD*)	0.15 (0.39)		0.12 (0.35)		0.11 (0.33)	
Residual variance (*SD*)	0.09 (0.31)		0.28 (0.53)		0.20 (0.45)	
−2log likelihood	519.7		809.6		690.2	

We controlled for eight covariates (age, education, income, subjective socioeconomic status, religion, length of the current relationship, attitude toward getting married, and negative interaction) in consideration of the differences between the intervention group and the comparison group that may exist after IPTW. See online Supplementary Table 5 and Table 6 for the results including all covariates.

^*^p < 0.05; ** *p* < 0.01; ***p < 0.001

^a^0 = no-intervention comparison group and 1 = intervention group

^b^0 = pretest and 1 = posttest

For women, the intervention-by-time interaction terms were statistically significant for marital readiness (*B* = 0.56, *p* < 0.001), relationship confidence (*B* = 0.49, *p* < 0.001), and relationship satisfaction (*B* = 0.25, *p* < 0.001). Simple slope analyses indicated that the pre-to-post changes in these three outcome variables differed significantly between the intervention group and the comparison group. In the intervention group, posttest scores were significantly higher than the pretest scores for marital readiness (*B* = 0.48, *p* < 0.001), relationship confidence (*B* = 0.31, *p* < 0.001), and relationship satisfaction (*B* = 0.12, *p* < 0.01), reflecting the intended outcomes. In contrast, the no-intervention comparison group demonstrated significant declines in marital readiness (*B* = − 0.08, *p* < 0.01), relationship confidence (*B* = − 0.16, *p* < 0.001), and relationship satisfaction (*B* = − 0.13, *p* < 0.001). As displayed in Fig. 2, our results suggest that S-PEP effectively enhanced marital readiness, relationship confidence, and relationship satisfaction for women.

For men, intervention-by-time interactions were significant for marital readiness (*B* = 0.42, *p* < 0.001) and relationship satisfaction (*B* = 0.19, *p* < 0.05), but no significant interaction effect was found for relationship confidence. Simple slope analyses revealed that, in the intervention group, posttest scores for marital readiness were significantly higher than the pretest scores (*B* = 0.32, *p* < 0.001). However, changes in relationship confidence (*B* = 0.05, *p* = 0.52) and relationship satisfaction (*B* = 0.04, *p* = 0.50) were not significant. In the no-intervention comparison group, all outcome variables showed significant decreases from pretest to posttest: marital readiness (*B* = − 0.10, *p* < 0.001), relationship confidence (*B* = − 0.16, *p* < 0.001), and relationship satisfaction (*B* = − 0.17, *p* < 0.001). As illustrated in Fig. 2, the results indicate that S-PEP was effective in increasing men’s marital readiness.

## Discussion

This study was an initial examination of whether S-PEP was effective in improving marital readiness, relationship confidence, and relationship satisfaction among Korean premarital women and men when the program was delivered via VC in the early COVID-19 period. Because of the infeasibility of conducting RCTs during this period, we recruited an intervention group and a no-intervention comparison group separately and conducted IPTW to adjust for the nonequivalence between the two groups. Using the weighted samples, our linear mixed models indicated that participation in VC-delivered S-PEP was associated with increased marital readiness for both premarital women and men, as well as greater relationship confidence and satisfaction for women.

The notable improvement in marital readiness, our primary outcome measure, suggests that S-PEP achieved the objective of improving marital readiness by equipping premarital couples with knowledge and skills related to major domains of marriage. S-PEP also provided opportunities for couples to engage in dialogue about their future married life before they tied the knot. Our results are consistent with previous literature reporting that premarital education improved marital readiness for US couples (McGeorge & Carlson, 2006). Our findings also imply that community-based public service providers can help premarital individuals and couples prepare for a healthy marriage by delivering preventive group interventions via VC. Although we examined the immediate effects of S-PEP on marital readiness, the positive changes found in this study are likely to contribute to preventing marital distress once the couples are married.

As for relationship quality, we found that participation in S-PEP was associated with increases in relationship confidence and relationship satisfaction for women. These findings are in line with a substantial number of studies reporting that CRE is effective in increasing the quality of couples’ relationships (Doss et al., 2016; Hawkins et al., 2022). However, the changes in men’s relationship confidence and relationship satisfaction were not significant in this study. We speculate that this result may be attributed to the fact that men’s levels of relationship confidence and relationship satisfaction were already high at pretest, leaving limited room for improvement after the intervention. A previous study examining the longitudinal trajectory of relationship confidence also found that individuals with initially higher levels of relationship confidence tended to experience only a slight increase in relationship confidence over time (Johnson et al., 2020). Additionally, S-PEP may have helped men reduce unrealistic optimism about their relationships and fostered realistic expectations, which are challenging to capture through variables like relationship confidence and satisfaction.

The significant decreases observed in all dependent variables for both women and men in the no-intervention comparison group may be partially explained by the timing of data collection, which occurred during the COVID-19 pandemic. Research has shown that the pandemic negatively affected couple relationships (Luetke et al., 2020; Pietromonaco & Overall, 2021). Even over short periods, relationship satisfaction tended to decline during this time (Martin et al., 2024). Thus, the unique challenges of the pandemic may have contributed to the declines observed in the comparison group, independent of the intervention effects.

The results of this study show that VC can be an effective method to deliver group-based preventive interventions for couples. Our findings extend previous studies that have only examined self-directed online CRE (Knopp et al., 2021; Megale et al., 2022) and individual virtual coaching and therapy (Dadds et al., 2019; Estrada et al., 2019; Marhefka et al., 2013). CRE via VC is a promising alternative for couples who have limited access to in-person CRE or couples who prefer virtual interventions. Given that synchronous human interaction via video has become part of everyday life especially for young people, VC-based youth relationship education including premarital education is suitable for today’s young adults. It is expected that VC will remain an important medium of communication long after the pandemic (Müller & Wittmer, 2023). Technological advancements will also improve the effectiveness of VC.

It is worth noting that VC-delivered CRE has both strengths and limitations. Common reasons that couples do not attend in-person CRE are busy schedules, geographic distance, and reluctance to disclose private information to other participants (Wilson & Halford, 2008). Self-directed online CRE has been a welcome solution because couples can attend sessions regardless of the time and place without privacy concerns. Compared to self-directed online CRE, the crucial strength of VC-delivered CRE is synchronous interaction among facilitators and participants, which promotes program effectiveness. However, real-time virtual interaction is an advantage only when couples are willing to interact with others. Some couples may still prefer interventions without interaction, particularly in Korea where people are relatively hesitant to mingle with strangers. In addition, VC sessions require attendance at a specified time, which could be a barrier to couples with time restraints. Access to high-speed Internet for VC could also be an issue for couples with limited resources. Therefore, we suggest that practitioners evaluate the strengths and limitations of each delivery mode (i.e., in-person CRE, self-directed online CRE, and VC-delivered CRE) and make informed decisions about the best medium of delivery. It is also critical to develop instructional strategies and teaching materials that maximize the advantages of each delivery mode and to strengthen practitioners’ instructional competency so they can effectively educate couples in the selected delivery context.

This study has important limitations as an initial attempt to examine the effectiveness of S-PEP via VC during the early COVID-19 period. First, we used a non-randomized controlled trial in that the intervention and comparison groups were recruited separately. Although IPTW helped us reduce the disadvantages of a nonequivalent comparison group design, IPTW does not adjust for group differences that may be due to unmeasured covariates. In addition, there was a 2-month gap between data collection from the intervention group and from the comparison group. Although we did not witness dramatic changes in the number of COVID-19 cases and the level of social distancing between the two months, the prolonged pandemic might have impacted the comparison group.

Second, we could not separate the effectiveness of S-PEP and the effectiveness of VC in our results because we tested the effectiveness of S-PEP via VC compared to no intervention. It would have been ideal if we had compared three groups—in-person S-PEP, VC-delivered S-PEP, and no-intervention—but unfortunately, the in-person delivery of S-PEP was restricted during the initial stages of the COVID-19 pandemic when we collected the data. Given that the Headquarters of Seoul Family Centers started to implement pre- and posttests of S-PEP in 2024, we expect that the effectiveness of in-person S-PEP will be tested soon. In addition, we suggest that future research compare the effectiveness of multiple modes of online delivery including VC, live streaming, self-directed web-based learning, and hybrid approaches that combine in-person and virtual interventions.

Finally, this study was limited because we did not examine the long-term effects of S-PEP and the potential moderators that may identify differences in the effectiveness of S-PEP. Our data were restricted to a pretest and posttest due to the short-term nature of our research project. Because the ultimate goal of premarital education is to prevent marital distress once the couples are married, we suggest that future research track couples for a longer duration beyond the honeymoon period when marital distress tends to be more common. Additionally, future research should test whether the effects of S-PEP differed depending on individual and couple characteristics. In the present study, these factors are simply controlled for, but numerous Western studies have shown that CRE is more effective for some couples because of their personal and relational resources (Job et al., 2017). Individual characteristics such as gender and mental health and couple characteristics such as relationship distress may have moderated the effect of VC-delivered S-PEP in this study. The effects may also vary depending on the type of smart devices used to participate in the program. A recent Korean study found that participating in VC-delivered parenting education using computers was associated with greater changes compared to participation using smart phones and tablets (Lee et al., 2021). Future research should examine what moderates the effects of CRE via VC.

Despite the limitations, this study contributes to the literature by providing initial evidence that VC can be an effective platform for group-based preventive interventions. In the present study, a VC-delivered premarital education program was effective in improving marital readiness and relationship quality among Korean premarital women and men when community-based public agencies delivered the curriculum. VC can help practitioners synchronously interact with individuals and couples who are not able to participate in in-person interventions due to physical and geographical constraints. More research is needed to examine the efficacy and effectiveness of VC-delivered preventive interventions.

## Supplementary Information

Below is the link to the electronic supplementary material.

Supplementary file1 (DOCX 67 KB)

## Data Availability

The data that support the findings of this study are available upon request.
