# Supplementary material for: Videoconferencing Delivery of the Seoul Premarital Education Program During COVID-19: A Quasi-experimental Study Using Inverse Probability of Treatment Weighting
Source: Prev Sci. 2025 Jan 6;27(1):131–43. doi: 10.1007/s11121-024-01761-z (PMC12906565; doi:10.1007/s11121-024-01761-z)
Supplement: Supplementary file 1 — Supplementary file1 (DOCX 67 KB) [file 11121_2024_1761_MOESM1_ESM.docx]

**Supplementary Table 1**

*Covariates Included in Propensity Score Matching*

| Variable | Measurement for propensity score matching |
| --- | --- |
| Age | 1 = 20-29 years old, 2 = 30-39 years old, 3 = 40-49 years old |
| Education | 1 = high school graduate or lower, 2 = some college, 3 = bachelor’s degree, 4 = graduate degree |
| Monthly income | Assessed at 11 levels (1 = Less than 1,000,000 KRW, 2 = 1,000,000–2,000,000 KRW; 10 = 9,000,000–10,000,000 KRW, 11 = More than 10,000,000 KRW); The median value in each level was adjusted using natural log transformation. |
| Subjective SES | 1 = low, 2 = middle-low, 3 = middle, 4 = middle-high, 5 = high |
| Religion | Dummy, 1 = have a religion |
| Length of current relationship | Years of relationship with the current partner |
| Positive attitude toward getting married | The extent of agreement with the statement, “It’s better to marry someone than staying single” (response categories: 1–5) |
| Negative couple interaction | An arithmetic mean score of 4 items (Negative Interaction Scale; Stanley et al., 2004; range = 1 to 5; Cronbach’s alpha at pretest = .81–.84) |

*Note*. All matching covariates were measured at pretest.

**Supplementary Table 2**

*Intercorrelations for Study Variables for Women (*N *= 291)*

| Variable | 1 |  | 2 |  | 3 |  | 4 |  | 5 |  | 6 |  | 7 |  | 8 |  | 9 |  | 10 |  | 11 |  | 12 |  | 13 |  |
| --- | --- | --- | --- | --- | --- | --- | --- | --- | --- | --- | --- | --- | --- | --- | --- | --- | --- | --- | --- | --- | --- | --- | --- | --- | --- | --- |
| 1. Age | – |  |  |  |  |  |  |  |  |  |  |  |  |  |  |  |  |  |  |  |  |  |  |  |  |  |
| 1. Education | .05 |  | – |  |  |  |  |  |  |  |  |  |  |  |  |  |  |  |  |  |  |  |  |  |  |  |
| 1. Income | .29 | ^***^ | .15 | ^**^ | – |  |  |  |  |  |  |  |  |  |  |  |  |  |  |  |  |  |  |  |  |  |
| 1. Subjective SES | .05 |  | .31 | ^***^ | .26 | ^***^ | – |  |  |  |  |  |  |  |  |  |  |  |  |  |  |  |  |  |  |  |
| 1. Has a religion | -.03 |  | .17 | **^**^** | .10 |  | .07 |  | – |  |  |  |  |  |  |  |  |  |  |  |  |  |  |  |  |  |
| 1. Length of relationship (year) | .00 |  | -.02 |  | -.06 |  | -.12 | **^*^** | .03 |  | – |  |  |  |  |  |  |  |  |  |  |  |  |  |  |  |
| 1. Marriage importance | .05 |  | .10 |  | .08 |  | .16 | **^**^** | -.00 |  | -.08 |  | – |  |  |  |  |  |  |  |  |  |  |  |  |  |
| 1. Negative interaction | -.03 |  | .05 |  | -.03 |  | -.11 |  | .10 |  | .16 | **^**^** | -.03 |  | – |  |  |  |  |  |  |  |  |  |  |  |
| 1. Marital readiness (pre) | -.11 |  | .03 |  | -.02 |  | .17 | **^**^** | -.02 |  | .09 |  | .08 |  | -.21 | **^***^** | – |  |  |  |  |  |  |  |  |  |
| 1. Marital readiness (post) | -.02 |  | .07 |  | -.04 |  | .18 | **^**^** | -.02 |  | -.03 |  | .15 | **^**^** | -.16 | **^**^** | .66 | **^***^** | – |  |  |  |  |  |  |  |
| 1. Marital confidence (pre) | -.07 |  | .01 |  | .01 |  | .19 | **^***^** | .04 |  | .02 |  | .18 | **^**^** | -.38 | **^***^** | .48 | **^***^** | .34 | ^***^ | – |  |  |  |  |  |
| 1. Marital confidence (post) | .01 |  | .03 |  | -.03 |  | .15 | **^**^** | .04 |  | .03 |  | .22 | **^***^** | -.37 | **^***^** | .34 | **^***^** | .52 | ^***^ | .67 | ^***^ | – |  |  |  |
| 1. Marital satisfaction (pre) | -.07 |  | .00 |  | .01 |  | .17 | **^**^** | .00 |  | -.08 |  | .16 | **^**^** | -.50 | **^***^** | .51 | **^***^** | .40 | ^***^ | .73 | ^***^ | .64 | ^***^ | – |  |
| 1. Marital satisfaction (post) | -.04 |  | .05 |  | -.01 |  | .15 | **^*^** | .03 |  | -.06 |  | .18 | **^**^** | -.42 | **^***^** | .33 | **^***^** | .44 | ^***^ | .55 | ^***^ | .77 | ^***^ | .72 | ^***^ |

*Note*. Correlations were computed using a balanced sample obtained through IPTW.

**p* < 0.05; ***p* < 0.01; ****p* < 0.001

**Supplementary Table 3**

*Intercorrelations for Study Variables for Men (*N *= 228)*

| Variable | 1 |  | 2 |  | 3 |  | 4 |  | 5 |  | 6 |  | 7 |  | 8 |  | 9 |  | 10 |  | 11 |  | 12 |  | 13 |  |
| --- | --- | --- | --- | --- | --- | --- | --- | --- | --- | --- | --- | --- | --- | --- | --- | --- | --- | --- | --- | --- | --- | --- | --- | --- | --- | --- |
| 1. Age | – |  |  |  |  |  |  |  |  |  |  |  |  |  |  |  |  |  |  |  |  |  |  |  |  |  |
| 1. Education | .15 | ^*^ | – |  |  |  |  |  |  |  |  |  |  |  |  |  |  |  |  |  |  |  |  |  |  |  |
| 1. Income | .25 | ^***^ | .23 | ^***^ | – |  |  |  |  |  |  |  |  |  |  |  |  |  |  |  |  |  |  |  |  |  |
| 1. Subjective SES | .08 |  | .36 | ^***^ | .29 | ^***^ | – |  |  |  |  |  |  |  |  |  |  |  |  |  |  |  |  |  |  |  |
| 1. Has a religion | -.02 |  | .07 |  | -.04 |  | -.04 |  | – |  |  |  |  |  |  |  |  |  |  |  |  |  |  |  |  |  |
| 1. Length of relationship (year) | .03 |  | -.11 |  | -.11 |  | -.17 | **^**^** | .06 |  | – |  |  |  |  |  |  |  |  |  |  |  |  |  |  |  |
| 1. Marriage importance | -.13 |  | -.01 |  | -.07 |  | .09 |  | .14 | ***** | -.18 | ****** | – |  |  |  |  |  |  |  |  |  |  |  |  |  |
| 1. Negative interaction | .01 |  | .05 |  | .08 |  | .07 |  | .05 |  | .13 |  | -.02 |  | – |  |  |  |  |  |  |  |  |  |  |  |
| 1. Marital readiness (pre) | -.10 |  | -.01 |  | .02 |  | .03 |  | .03 |  | .05 |  | .07 |  | -.7 | **^**^** | – |  |  |  |  |  |  |  |  |  |
| 1. Marital readiness (post) | .02 |  | .04 |  | .05 |  | .08 |  | .03 |  | .03 |  | .05 |  | -.17 | **^*^** | .66 | **^***^** | – |  |  |  |  |  |  |  |
| 1. Marital confidence (pre) | -.09 |  | -.11 |  | -.01 |  | .06 |  | -.02 |  | -.03 |  | .19 | **^**^** | -.36 | **^***^** | .49 | **^***^** | .39 | ^***^ | – |  |  |  |  |  |
| 1. Marital confidence (post) | .02 |  | .02 |  | .02 |  | .11 |  | .04 |  | -.05 |  | .17 | **^**^** | -.33 | **^***^** | .35 | **^***^** | .51 | ^***^ | .49 | ^***^ | – |  |  |  |
| 1. Marital satisfaction (pre) | -.16 | **^*^** | -.09 |  | -.10 |  | -.02 |  | .03 |  | -.03 |  | .24 | **^***^** | -.48 | **^***^** | .45 | **^***^** | .37 | ^***^ | .68 | ^***^ | .47 | ^***^ | – |  |
| 1. Marital satisfaction (post) | -.00 |  | .06 |  | -.03 |  | .11 |  | -.01 |  | -.07 |  | .24 | **^***^** | -.37 | **^***^** | .28 | **^***^** | .45 | ^***^ | .42 | ^***^ | .77 | ^***^ | .58 | ^***^ |

*Note*. Correlations were computed using a balanced sample obtained through IPTW.

**p* < 0.05; ***p* < 0.01; ****p* < 0.001

**Supplementary Table 4**

*Covariates Descriptive Statistics and Standardized Mean Differences between the Intervention and Comparison Groups Before and After IPTW*

|  | Women | | | | |  | Men | | | | |
| --- | --- | --- | --- | --- | --- | --- | --- | --- | --- | --- | --- |
|  | Intervention  (*n* = 114) | Comparison  (*n* = 177) | *SMD* | | Variance  ratio |  | Intervention  (*n* = 82) | Comparison  (*n* = 146) | *SMD* | | Variance ratio |
| Variable | *n* (%) | *n* (%) | Initial  sample | Weighted sample |  |  | *n* (%) | *n* (%) | Initial  sample | Weighted sample |  |
| Age |  |  |  |  |  |  |  |  |  |  |  |
| 20-29 | 50 (43.9) | 101 (57.1) | 0.25 | 0.19 | 0.93 |  | 21 (25.6) | 59 (40.4) | 0.32 | 0.20 | 0.94 |
| 30-39 | 64 (56.1) | 76 (42.9) |  |  |  |  | 61 (74.4) | 87 (59.6) |  |  |  |
| Education |  |  |  |  |  |  |  |  |  |  |  |
| High school graduate or lower | 2 (1.8) | 9 (5.1) | 0.26 | 0.16 | 0.92 |  | 4 (4.9) | 17 (11.6) | 0.24 | 0.13 | 0.56 |
| Some college | 11 (9.6)) | 21 (11.9) |  |  |  |  | 3 (3.7) | 14 (9.6) |  |  |  |
| Bachelor’s degree | 81 (71.1) | 129 (72.9) |  |  |  |  | 67 (81.7) | 98 (67.1) |  |  |  |
| Graduate degree | 20 (17.5) | 18 (10.2) |  |  |  |  | 8 (9.8) | 17 (11.6) |  |  |  |
| Subjective SES |  |  |  |  |  |  |  |  |  |  |  |
| Low | 6 (5.3) | 11 (6.2) | 0.24 | 0.16 | 1.31 |  | 5 (601) | 17 (11.6) | 0.31 | 0.19 | 0.88 |
| Middle-low | 28 (24.6) | 52 (29.4) |  |  |  |  | 23 (28.0) | 55 (37.7) |  |  |  |
| Middle | 52 (45.6) | 88 (49.7) |  |  |  |  | 38 (46.3) | 56 (38.4) |  |  |  |
| Middle-high | 23 (20.2) | 25 (14.1) |  |  |  |  | 15 (18.3) | 16 (11.0) |  |  |  |
| High | 5 (4.4) | 1 (0.6) |  |  |  |  | 1 (1.2) | 2 (1.4) |  |  |  |
| Has a religion | 43 (37.7) | 61 (34.5) | .07 | .02 | 1.02 |  | 38 (46.3) | 52 (35.6) | .22 | .12 | 1.04 |
|  | *M* (*SD*) | *M* (*SD*) |  |  |  |  | *M* (*SD*) | *M* (*SD*) |  |  |  |
| Age | 30.31 (2.88) | 29.24 (3.65) | – | – | – |  | 32.37 (3.79) | 30.70 (4.13) | – | – | – |
| Income | 3.04 (1.17) | 2.82 (1.02) | 0.21 | 0.11 | 0.80 |  | 3.78 (1.69) | 3.20 (1.40) | 0.38 | 0.24 | 0.81 |
| Subjective SES (1-5) | 2.94 (0.91) | 2.73 (0.80) | – | – | – |  | 2.80 (0.85) | 2.53 (0.89) | – | – | – |
| Length of the relationship (year) | 2.87 (2.30) | 3.56 (2.62) | -0.28 | -0.18 | 0.85 |  | 2.80 (2.24) | 3.12 (2.18) | -0.15 | -0.08 | 1.09 |
| Marriage importance | 3.82 (1.01) | 3.91 (1.01) | -0.08 | -0.08 | 1.01 |  | 4.22 (0.98) | 4.03 (0.96) | 0.19 | 0.12 | 1.14 |
| Negative interaction | 2.18 (0.86) | 1.92 (0.84) | 0.30 | 0.15 | 0.87 |  | 2.25 (0.84) | 2.10 (0.89) | 0.17 | 0.09 | 0.85 |
| SMD = standardized mean difference | | | | | | | | | | | |

**Supplementary Table 5**

*Fixed Effects Results from the Linear Mixed Models Including Covariates for Women (*N *= 291)*

|  | Marital  readiness | | |  | Relationship confidence | | |  | Relationship satisfaction | | |
| --- | --- | --- | --- | --- | --- | --- | --- | --- | --- | --- | --- |
| Variable | *B* | | *SE* |  | *B* | | *SE* |  | *B* | | *SE* |
| Fixed effects |  |  |  |  |  |  |  |  |  |  |  |
| Intercept | 3.98 | ^***^ | 0.77 |  | 4.71 | ^***^ | 0.92 |  | 4.66 | ^***^ | 0.80 |
| Intervention ^a^ | -0.07 |  | 0.06 |  | -0.14 |  | 0.08 |  | -0.01 |  | 0.07 |
| Time ^b^ | -0.08 | ^**^ | 0.03 |  | -0.18 | ^***^ | 0.04 |  | -0.13 | ^***^ | 0.03 |
| Intervention by time | 0.56 | ^***^ | 0.05 |  | 0.49 | ^***^ | 0.07 |  | 0.25 | ^***^ | 0.06 |
| Age | -0.10 |  | 0.06 |  | -0.05 |  | 0.07 |  | -0.11 |  | 0.06 |
| Education | 0.01 |  | 0.05 |  | -0.02 |  | 0.06 |  | -0.00 |  | 0.05 |
| Income | -0.04 |  | 0.05 |  | -0.06 |  | 0.07 |  | -0.02 |  | 0.06 |
| SES | 0.11 | ^**^ | 0.04 |  | 0.13 | ^**^ | 0.04 |  | 0.09 | ^*^ | 0.04 |
| Religion | -0.05 |  | 0.06 |  | 0.08 |  | 0.07 |  | 0.07 |  | 0.06 |
| Length of the relationship | 0.02 |  | 0.01 |  | 0.04 | ^**^ | 0.01 |  | 0.01 |  | 0.01 |
| Attitudes toward getting married | 0.06 | ^*^ | 0.03 |  | 0.13 | ^***^ | 0.03 |  | 0.10 | ^***^ | 0.03 |
| Negative interaction | -0.11 | ^***^ | 0.03 |  | -0.31 | ^***^ | 0.07 |  | -0.32 | ^***^ | 0.03 |
| Random effects |  |  |  |  |  |  |  |  |  |  |  |
| Intercept variance (*SD*) | 0.18 (0.43) | | |  | 0.24 (0.49) | | |  | 0.18 (.43) | | |
| Residual variance (*SD*) | 0.10 (0.32) | | |  | 0.21 (0.45) | | |  | 0.15 (.38) | | |
| -2log likelihood | 707.5 | | | | 1020.0 | | | | 839.1 | | |

^a^ 0 = no-intervention comparison group and 1 = intervention group. ^b^ 0 = pretest and 1 = posttest.

**p* < 0.05; ***p* < 0.01; ****p* < 0.001

**Supplementary Table 6**

*Fixed Effects Results from the Linear Mixed Models Including Covariates for Men (*N *= 228)*

|  | Marital readiness | | |  | Relationship confidence | | |  | Relationship satisfaction | | |
| --- | --- | --- | --- | --- | --- | --- | --- | --- | --- | --- | --- |
|  |  | | |  |  | | |  |  | | |
| Variable | *B* | | *SE* |  | *B* | | *SE* |  | *B* | | *SE* |
| Fixed effects |  |  |  |  |  |  |  |  |  |  |  |
| Intercept | 3.37 | ^***^ | 0.79 |  | 3.83 | ^***^ | 0.87 |  | 4.69 | ^***^ | 0.79 |
| Intervention ^a^ | 0.04 |  | 0.07 |  | 0.26 | ^**^ | 0.09 |  | 0.21 | ^**^ | 0.08 |
| Time ^b^ | -0.09 | ^**^ | 0.03 |  | -0.14 | ^**^ | 0.05 |  | -0.15 | ^***^ | 0.04 |
| Intervention by time | 0.42 | ^***^ | 0.06 |  | 0.19 |  | 0.10 |  | 0.19 | ^*^ | 0.08 |
| Age | -0.08 |  | 0.06 |  | -0.06 |  | 0.06 |  | -0.08 |  | 0.06 |
| Education | 0.01 |  | 0.04 |  | -0.06 |  | 0.05 |  | -0.01 |  | 0.04 |
| Income | 0.03 |  | 0.05 |  | 0.03 |  | 0.06 |  | -0.03 |  | 0.05 |
| SES | 0.03 |  | 0.04 |  | 0.09 | ^*^ | 0.04 |  | 0.05 |  | 0.04 |
| Religion | 0.02 |  | 0.06 |  | 0.01 |  | 0.07 |  | -0.02 |  | 0.06 |
| Length of the relationship | 0.02 |  | 0.01 |  | 0.02 |  | 0.02 |  | 0.02 |  | 0.01 |
| Attitudes toward getting married | 0.03 |  | 0.03 |  | 0.12 | ^**^ | 0.03 |  | 0.14 | ^***^ | 0.03 |
| Negative interaction | -0.11 | ^**^ | 0.03 |  | -0.26 | ^***^ | 0.04 |  | -0.29 | ^***^ | 0.03 |
| Random effects |  |  |  |  |  |  |  |  |  |  |  |
| Intercept variance (*SD*) | 0.15 (0.39) | | |  | 0.12 (0.35) | | |  | 0.11 (0.33) | | |
| Residual variance (*SD*) | 0.09 (0.31) | | |  | 0.28 (0.53) | | |  | 0.20 (0.45) | | |
| -2log likelihood | 519.7 | | | | 809.6 | | | | 690.2 | | |

^a^ 0 = no-intervention comparison group and 1 = intervention group. ^b^ 0 = pretest and 1 = posttest.

**p* < 0 .05; ***p* < 0.01; ****p* < 0.001
